# Efficacy of a two-tiered trauma team activation protocol in a Norwegian trauma centre

**DOI:** 10.1002/bjs.7794

**Published:** 2012-02

**Authors:** M Rehn, H M Lossius, K E Tjosevik, M Vetrhus, O Østebø, T Eken, Rogaland Trauma System Study Collaborating Group

**Affiliations:** 1 Department of Research, Norwegian Air Ambulance FoundationDrøbak, Norway; 2 Akershus University HospitalLørenskog, Norway; 3 Department of Surgical Sciences, University of BergenBergen, Norway; 4 Acute Clinic, Stavanger University HospitalStavanger, Norway; 5 Department of Surgery, Stavanger University HospitalStavanger, Norway; 6 Department of Anaesthesiology, Oslo University Hospital UllevålOslo, Norway

## Abstract

**Background:**

A registry-based analysis revealed imprecise informal one-tiered trauma team activation (TTA) in a primary trauma centre. A two-tiered TTA protocol was introduced and analysed to examine its impact on triage precision and resource utilization.

**Methods:**

Interhospital transfers and patients admitted by non-healthcare personnel were excluded. Undertriage was defined as the fraction of major trauma victims (New Injury Severity Score over 15) admitted without TTA. Overtriage was the fraction of TTA without major trauma.

**Results:**

Of 1812 patients, 768 had major trauma. Overall undertriage was reduced from 28·4 to 19·1 per cent (*P* < 0·001) after system revision. Overall overtriage increased from 61·5 to 71·6 per cent, whereas the mean number of skilled hours spent per overtriaged patient was reduced from 6·5 to 3·5 (*P* < 0·001) and the number of skilled hours spent per major trauma victim was reduced from 7·4 to 7·1 (*P* < 0·001). Increasing age increased risk for undertriage and decreased risk for overtriage. Falls increased risk for undertriage and decreased risk for overtriage, whereas motor vehicle-related accidents showed the opposite effects. Patients triaged to a prehospital response involving an anaesthetist had less chance of both undertriage and overtriage.

**Conclusion:**

A two-tiered TTA protocol was associated with reduced undertriage and increased overtriage, while trauma team resource consumption was reduced. Registration number: NCT00876564 (http://www.clinicaltrials.gov). Copyright © 2011 British Journal of Surgery Society Ltd. Published by John Wiley & Sons, Ltd.

Effective system to deal with local and distant trauma

## Introduction

Early recognition of major trauma enables emergency medical services (EMS) to accurately triage and transport injured patients to an appropriate hospital. Field triage, however, remains a challenge due to occult injuries, the unpredictable evolution of symptoms and complexities of evaluating patients in difficult circumstances. A combined literature review and US national expert panel consensus resulted in ‘Guidelines for Field Triage of Injured Patients’^1^, ^2^. This presented a stepwise evaluation of trauma victims for physiological instability, obvious anatomical injury, mechanism of injury and co-morbidity. The report recommended that tiered trauma care should be provided according to the probability of having sustained major trauma.

Norway is sparsely populated with weather-dependent and time-consuming patient transport. Some 50 Norwegian hospitals receive patients with major injuries, most with low admission rates^3^. In an attempt to optimize patient outcome^4^, immediate resuscitation is increasingly being delivered via multidisciplinary one-tiered trauma teams. However, several studies indicate a trend for imprecise activation of such teams^5^–^8^.

If patients with major injuries are deprived access to the possible benefits of immediate resuscitation and expert evaluation provided by a trauma team (undertriage), avoidable deaths may occur^9^. Conversely, if the trauma team attends patients with minor injuries (overtriage), scarce financial and human resources are consumed. To improve triage efficacy, a two-tiered trauma team activation (TTA) response has been recommended^1^. A full trauma team should attend patients suffering from obvious major injury, but a reduced trauma team may systematically evaluate patients where the extent of injury is unclear. A growing body of evidence suggests that a tiered response is safe and cost-effective^10^–^21^. The American College of Surgeons considers 5 per cent undertriage associated with 25–50 per cent overtriage as acceptable^22^. An unpublished registry-based analysis of the informal one-tiered TTA practice at Stavanger University Hospital (SUH) revealed unacceptably high undertriage and overtriage rates. For this reason, a two-tiered TTA protocol was developed and implemented at this trauma centre according to international recommendations^1^. The impact of this system revision on medical resource utilization and triage precision was evaluated using trauma registry data.

## Methods

SUH is a 630-bed primary trauma centre for a mixed rural/urban population of approximately 330 000 inhabitants and the trauma referral centre for an additional 120 000 people living in Rogaland county in southwestern Norway. The hospital admits each year approximately 140 adult and paediatric patients with a New Injury Severity Score^23^ (NISS) greater than 15^24^, ^25^. A hospital-based trauma registry has been fully operational since 2004. An Association for the Advancement of Automotive Medicine-certified Abbreviated Injury Scale (AIS) coder (a registered nurse) manually searches the hospital administrative data system for relevant patients (*Table* *1*) and annually codes data on approximately 360 patients.

**Table 1:** Inclusion and exclusion criteria for the Stavanger University Hospital trauma registry

Inclusion criteria	Exclusion criteria
Absolute criteria	Patients not fulfilling the absolute
Activated trauma team	criteria
Penetrating injury to	*or*
Head	Isolated fracture with skin injury
Neck	(AIS 1) in
Trunk	Upper extremity
Extremities proximal to	Lower extremity
knee or elbow	Floor of orbita
Relative criteria	Chronic subdural haematoma
ISS ≥ 10	Drowning, inhalation injury,
NISS > 15*	asphyxia-related injury (hanging,
	strangulation)
	Secondary admission to SUH
	> 24 h after injury

* After implementing the Utstein template for uniform reporting of data following major trauma. AIS, Abbreviated Injury Scale; ISS, Injury Severity Score; NISS, New Injury Severity Score; SUH, Stavanger University Hospital.

Prehospital emergency care in the SUH catchment area is provided by on-call general practitioners, vehicle ambulance units staffed by paramedics and emergency medical technicians, and anaesthetist-manned rapid response cars and helicopters^26^. Until February 2009, the hospital practised informal activation of a one-tiered 13-personnel multidisciplinary trauma team.

The Rogaland Trauma System Study Group was established by SUH in 2008 in cooperation with the Norwegian Air Ambulance Foundation research department. The group comprised clinical representatives from the emergency department, dispatch, surgery, anaesthesiology, and ground and air ambulance units in addition to researchers. They developed guidelines on field triage and TTA based on available evidence^1^, ^5^ and multidisciplinary consensus on optimal local practice. EMS providers were empowered to assign patients into two tiers of TTA according to field triage criteria (*Table* *2*). Activation of the full multidisciplinary trauma team was based on physiological or anatomical criteria. The purpose of the full team was to provide immediate resuscitation and rapid evaluation, and initiation of definitive care. A reduced team was initiated in patients not meeting the criteria for the full team but when there was either one mechanism of injury or one co-morbidity criterion present (*Table* *3*). The purpose of the reduced team was rapidly to assess physiologically stable patients for occult injuries. When two or more mechanisms of injury or co-morbidity criteria were fulfilled the full team was activated. The reduced team was capable of rapid upgrading to a full team if potentially severe injures were detected. Both full and reduced teams were led by the same surgeon with a minimum of 2 years of experience in surgery and certified as an Advanced Trauma Life Support provider. The remaining team members had no formal competence requirements. Additional surgical subspecialty resources were available at the team leader's discretion.

**Table 2:** Triage criteria for tiered trauma team activation (full and reduced)

Full trauma team	Reduced trauma team
1. Physiology	5. Co-morbidity
1·1 RTS ≤ 11	5·1 Age > 60 years
1·2 GCS < 14	5·2 Age < 6 years
1·3 Respiratory rate < 9/min	5·3 Severe co-morbidity (e.g.
1·4 Respiratory rate > 25/min	COPD, congestive heart
1·5 *S*po_2_ < 90%	failure)
1·6 Intubated/attempted	5·4 Pregnancy
intubation	5·5 Increased risk of haemorrhage
1·7 Obvious massive	(anticoagulant drugs,
haemorrhage	coagulopathy)
1·8 Systolic blood pressure	
< 90 mmHg	6. Mechanism of injury
	6·1 Co-passenger killed
2. Anatomy	6·2 Entrapped person
2·1 Facial injury with risk for	6·3 Person ejected from
airway obstruction	vehicle/motorcycle
2·2 Flail chest	6·4 Pedestrian, cyclist run down
2·3 Suspected pneumothorax	at > 30 km/h or thrown up
2·4 Stab or gunshot wound	in the air
proximal to knee or elbow	6·5 Collision speed > 50 km/h
2·5 Suspected pelvic fracture	6·6 Deformed vehicle
2·6 Crushed, mangled or	compartment
amputated extremity	6·7 Airbag set off
2·7 Two or more long bone	6·8 Vehicle roll-over
fractures	6·9 Fall > 5 m (adults)
2·8 Open fracture with	6·10 Fall > 3 m (children)
ongoing haemorrhage	
2·9 Open skull fracture or	7. Interhospital transfer
impression fracture	7·1 Interhospital transfer and
2·10 Suspected spinal cord	< 24 h since time of injury
injury	
2·11 Burn injury (≥ grade II)	Note: If two or more criteria under
> 15% total body surface	list 5 or 6 are fulfilled, activate
area	full trauma team
3. Several patients	
3·1 Accident with several	
severely injured	
(suspected or confirmed)	
4. Upgrade to full trauma	
team	
4·1 When two or more criteria	
for reduced trauma	
team (list 5 or 6) are	
fulfilled	
4·2 When reduced trauma	
team finds a perceived	
stable patient to be	
unstable	

RTS, Revised Trauma Score; GCS, Glasgow Coma Scale; COPD, chronic obstructive pulmonary disease; *S*po_2_, oxygen saturation measured by pulse oximetry.

**Table 3:** Trauma team composition (full and reduced)

Full trauma team (13 members)	Reduced trauma team (4 members)
Team leader surgeon*	Team leader surgeon*
Orthopaedic surgeon†	Orthopaedic surgeon†
Theatre nurse	2 ED nurses
3 ED nurses	
Anaesthetist†	
Nurse anaesthetist	
Radiologist†	
2 radiographers	
Laboratory technician	
Orderly	

* Minimum of 2 years' experience with surgery and certified Advanced Trauma Life Support provider.

†No formal competence requirements. ED, emergency department.

The trauma registry was upgraded to prospectively collect data necessary to compare practice after introduction of the two-tiered guidelines. The guidelines were launched on 3 February 2009 under the direction of the Rogaland Trauma System Study Group. Throughout the implementation period, instructors addressed specific aspects of the system revision during educational outreach visits. Information posters and periodical newsletters were used to increase understanding and awareness of the system revision.

The trial was designed as a prospective interventional study utilizing SUH trauma registry data and was divided into an analysis of the ‘before’ period, which consisted of patients subject to the informal one-tiered practice (1 January 2004 to 31 December 2008), and an analysis of the ‘after’ period, which consisted of patients subject to the two-tiered TTA protocol (1 July 2009 to 31 December 2010). The implementation period (1 January 2009 to 30 June 2009) was excluded from the analysis.

Consecutive patients admitted to SUH during the study period who were registered in the SUH trauma registry and assigned one or more AIS codes were included if they had major trauma (NISS over 15) and/or had been triaged to meet the trauma team (*Table* *4*, groups *a*, *b* and *c*). The AIS 1998 catalogue was used for all patients^27^. Interhospital transfers to SUH and patients admitted by non-healthcare personnel were excluded. Survival status 30 days after injury^28^ was obtained from patient records and the Norwegian Population Registry. The Standards for Quality Improvement Reporting (SQUIRE)^29^, Standards for Reporting of Diagnostic Accuracy (STARD) statement^30^ and Strengthening the Reporting of Observational Studies in Epidemiology (STROBE) guidelines were used^31^.

**Table 4:** Injury severity and trauma team activation

	Major trauma	Not major trauma	Total
TTA	*a*	*b*	*a* + *b*
No TTA	*c*	*d*	*c* + *d*
Total	*a* + *c*	*b* + *d*	*n*

Sensitivity = *a*/(*a* + *c*); specificity = *d*/(*b* + *d*); positive predictive value (PPV) = *a*/(*a* + *b*); undertriage = 1 − sensitivity = *c*/(*a* + *c*); overtriage = 1 − PPV = *b*/(*a* + *b*). TTA, trauma team activation.

The Regional Committee for Medical and Health Research Ethics deemed the system revision to be a quality improvement initiative not in need of formal approval (2009/228-CAG). The Norwegian Social Science Data Services approved access to aggregate anonymous data on relevant patients in the hospital-based trauma registry (20 840 KS/LR). The study was registered in clinicaltrials.gov (NCT00876564).

### Statistical analysis

Patients were classified as major trauma victims if they had an NISS above 15^28^. The evaluation of triage precision was based on the assumption that all patients with major injury benefit from assessment by a trauma team upon arrival at hospital. Sensitivity was defined as the probability for major trauma victims to be assessed by a full and/or reduced trauma team. Undertriage was defined as the contrary event (1–sensitivity), the probability of not being examined by a trauma team (full and/or reduced) despite having a major injury. To calculate specificity and thereby the conventional definition of overtriage (1—specificity)^32^, the number of patients with minor injuries admitted without an activated trauma team (true negatives; group *d* in *Table* *4*) must be identified. As SUH annually treats a large number of patients (approximately 3400 subjects) with only minor injuries, the classical definition is of limited usefulness. This substantial and not easily definable group of patients is rarely considered in need of assessment by a trauma team, and would strongly bias a computation of overtriage based on specificity. Overtriage was therefore defined as the complement of the positive predictive value, 1 − PPV, where PPV represents the probability of a patient suffering from major trauma when the trauma team is activated (*Table* *4*)^33^. This is equivalent to the proportion of patients without major trauma among those who were triaged to a trauma team.

In addition to direct comparison of overtriage rates ‘before’ and ‘after’ system revision, skilled hours' expenditure on overtriage per major trauma victim was measured. For each member of the trauma team, 30 min per unnecessary activation was allocated (full trauma team, 13 members = 6·5 skilled hours; reduced trauma team, 4 members = 2 skilled hours; *Table* *3*).

Probability of survival was calculated using the Trauma Score—Injury Severity Score (TRISS) methodology^34^ with 1995 coefficients^35^. The *W* statistic^36^ (expressing excess survivors per 100 patients compared with TRISS model predictions) with 95 per cent confidence interval (c.i.) was used to compare outcomes from the two study periods^33^. Non-overlapping 95 per cent c.i. were considered to indicate significant differences in survival.

Categorical variables were compared with Fisher's exact test, whereas continuous variables were analysed using the Mann–Whitney *U* test. Assumed predictors of overtriage and undertriage were tested in a multiple logistic regression analysis. All data were analysed using STATA/SE™ version 10.1 (StataCorp LP, College Station, Texas, USA) and StatView version 5.0.1 (SAS Institute, Cary, North Carolina, USA). Statistical significance was assumed for *P* < 0·050.

## Results

During the study period (1 January 2004 to 31 December 2010), 2327 patients were entered in the SUH trauma registry. Some 364 injured patients who were transferred to SUH from other hospitals, admitted by non-healthcare personnel or admitted during the new TTA criteria implementation period (1 January 2009 to 30 June 2009) were excluded. A further 151 patients who had neither sustained major trauma nor been triaged to a trauma team (true-negatives) were also excluded. In total, 1812 patients met the inclusion criteria and were enrolled in the study. There was a missing probability of survival for seven patients and lack of documentation of TTA criteria in 123, but otherwise data were complete.

*Table* *5* shows population characteristics of included patients in the ‘before’ and ‘after’ study periods. Distribution of age and sex, proportion of accidents involving motor vehicles and the proportion of penetrating *versus* blunt injuries did not change significantly between the two study periods.

**Table 5:** Patients included in the ‘before’ and ‘after’ study periods

	Before	After	*P*†
Included patients (TTA and/or major trauma)	1255	557	
Age (years)*	31 (19–51)	34 (20–53)	0·280
Sex ratio (F:M)	354:901	155:402	0·910
Falls	273 (21·8)	164 (29·4)	0·001
Motor vehicle-related accidents	498 (39·7)	204 (36·6)	0·230
Dominant injury (penetrating:blunt)	58:1197 (4·8:95·2)	22:535 (3·9:96·1)	0·620
NISS*	12 (5–26)	8 (3–18)	< 0·001
Major trauma	585 (46·6)	183 (32·9)	< 0·001
Prehospital anaesthetist (yes:no)	737:518 (58·7:41·3)	271:286 (48·7:51·3)	< 0·001
TTA	1089 (86·8)	522 (93·7)	< 0·001
Deaths (unadjusted)	78 (6·2)	16 (2·9)	0·003

Values in parentheses are percentages unless otherwise stated;

* values are median (interquartile range). TTA, trauma team activation; NISS, New Injury Severity Score; major trauma, NISS > 15.

†Fisher's exact test for categorical variables; Mann–Whitney *U* test for continuous variables.

In the ‘after’ period, there was a significant increase in the proportion of traumas due to falls. The proportion of patients who met an anaesthetist before hospital decreased significantly and a higher proportion of the included patients had been triaged to receive a full or reduced trauma team. Median NISS score, proportion of patients with major trauma and number of deaths in ‘after’ patients were significantly lower.

Triage categories of included patients are shown in *Table* *6*. Among the 1255 patients included in the ‘before’ study period, 1089 (86·8 per cent) were triaged to a trauma team. In the ‘after’ study period, 522 of 557 patients (93·7 per cent) were triaged to a team, 232 to the full team and 290 to the reduced team.

**Table 6:** Triage categories and prehospital response types

	Before		After			
		
	TTA	Not TTA	TTA			Not TTA
				
	Total (MT:not MT)	Total (MT)	Total (MT:not MT)	Full team (MT:not MT)	Reduced team (MT:not MT)	Total (MT)
All	419:670	166	148:374	108:124	40:250	35
Prehospital anaesthetist	338:364	35	99:165	80:73	19:92	7
No prehospital anaesthetist	81:306	131	49:209	28:51	21:158	28

TTA, trauma team activation; MT, major trauma (New Injury Severity Score > 15).

### Undertriage and overtriage

In the ‘before’ period, 166 of the 585 patients with major trauma (28·4 per cent) were not triaged to a trauma team, and this fell to 35 of 183 (19·1 per cent) in the ‘after’ period (*P* < 0·001). There was a 41·2 per cent relative reduction in undertriage rate in responses without anaesthetists, whereas the decrease in the low rate of undertriage performed by prehospital anaesthetists was not significant.

The proportion of patients triaged to a trauma team who had not suffered major trauma increased from 670 of 1089 (61·5 per cent) in the ‘before’ study period to 374 of 522 (71·6 per cent) in the ‘after’ period (*P* < 0·001). The increase was most pronounced in prehospital responses with an anaesthetist, although responses without anaesthetists still had the highest rate (*Table* *7*).

**Table 7:** Changes in triage categories by prehospital response types

		Before (%)	After (%)	Absolute change (%)	Relative change (%)	P*
Undertriage	All	28·4	19·1	− 9·3	− 32·6	< 0·001
	Prehospital anaesthetist	9·4	6·6	− 2·8	− 29·6	0·155
	No prehospital anaesthetist	61·8	36·4	− 25·4	− 41·2	< 0·001
Overtriage, total	All	61·5	71·6	10·1	16·5	< 0·001
	Prehospital anaesthetist	51·9	62·5	10·6	20·5	0·001
	No prehospital anaesthetist	79·1	81·0	1·9	2·5	< 0·001
Overtriage, full team	All		53·4			
	Prehospital anaesthetist		47·7			
	No prehospital anaesthetist		64·6			
Overtriage, reduced team	All		86·2			
	Prehospital anaesthetist		82·9			
	No prehospital anaesthetist		88·3			

* Fisher's exact test.

The proportion of patients who had not suffered major trauma was particularly high in patients assigned to receive reduced teams (250 of 290, 86·2 per cent) compared with 124 of 232 (53·4 per cent) in patients triaged to receive full teams (*P* < 0·001) (*Table* *7*).

The mean number of skilled hours spent per overtriaged patient was reduced from 6·5 to 3·5 (*P* < 0·001), whereas the number of skilled hours spent per major trauma victim was reduced from 7·4 to 7·1 (*P* < 0·001).

**Fig. 1:**
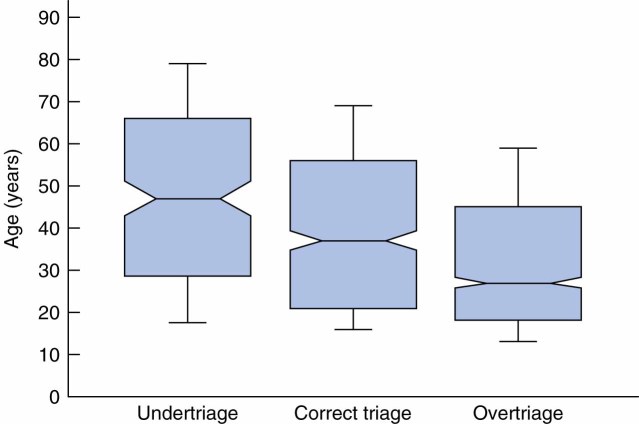
Relationship between patient age and triage category. Box plots depict medians and interquartile ranges; whiskers represent 10th and 90th percentiles. Note non-overlapping 95 per cent confidence intervals for medians (notches)

After initially finding an association between age and mistriage (*Fig.* *1*), age was included as an independent variable in the logistic regression models, along with sex, fall, motor vehicle-related accident, prehospital response type (with *versus* without anaesthetist) and study period (‘after’ *versus* ‘before’). Results are shown in *Table* *8*.

**Table 8:** Odds ratios for undertriage and overtriage in the logistic regression model

	Odds ratio	*P*
Undertriage*		
Age (per decade)	1·28 (1·18, 1·39)	< 0·001
Sex (F *versus* M)	1·26 (0·86, 1·87)	0·241
Fall (yes *versus* no)	2·46 (1·71, 3·55)	< 0·001
Motor vehicle-related	0·09 (0·04, 0·18)	< 0·001
accident (yes *versus* no)		
Prehospital anaesthetist	0·16 (0·11, 0·24)	< 0·001
(yes *versus* no)		
Period (after *versus* before)	0·26 (0·17, 0·40)	< 0·001
Overtriage*		
Age (per decade)	0·79 (0·75, 0·83)	< 0·001
Sex (F *versus* M)	1·38 (1·10, 1·74)	0·006
Fall (yes *versus* no)	0·67 (0·52, 0·87)	0·003
Motor vehicle-related	2·07 (1·64, 2·62)	< 0·001
accident (yes *versus* no)		
Prehospital anaesthetist	0·55 (0·45, 0·68)	< 0·001
(yes *versus* no)		
Period (after *versus* before)	1·97 (1·57, 2·46)	< 0·001

Values in parentheses are 95 per cent confidence intervals.

* Overall model *R*^2^ for undertriage 0·101; for overtriage 0·291.

All but one variable showed consistent and significant effects on triage. Increasing age clearly increased risk for undertriage and decreased risk for overtriage. For mechanisms of injury, falls showed increased risk for undertriage and decreased risk for overtriage, whereas motor vehicle-related accidents showed the opposite effects. Patients triaged by the emergency medical communication centre to a prehospital response involving an anaesthetist had reduced risk for both undertriage and overtriage. In the ‘after’ study period, risk for undertriage was reduced whereas risk for overtriage was increased. In this multiple logistic regression model, sex showed inconsistent effects on triage, possibly owing to a correlation between female sex, advanced age and trauma due to falls.

Analysis of individual TTA criteria in the ‘after’ study period for usage and overtriage showed that for reduced teams mechanism of injury criteria were associated with 89·4 per cent overtriage and co-morbidity criteria with 68 per cent overtriage (*Table* *9*). Criteria were undocumented for 70 (24·5 per cent) of 286 reduced teams (79 per cent overtriage). For full teams, criteria pertaining to physiology were associated with 41 per cent overtriage, and criteria depicting anatomical injury with 59 per cent overtriage. Criteria were undocumented for 53 (23·1 per cent) of 229 full teams (62 per cent overtriage). Upgraded TTA due to the patient being unstable was applied to five patients of whom one had suffered minor injuries only (20 per cent overtriage). Four patients had falls and one was involved in a motor vehicle accident.

**Table 9:** Trauma team activation criteria in the ‘after’ period: frequency and overtriage

	*n*	Overtriage
Full team		
Physiology		
RTS ≤ 11	18	4 (22)
GCS < 14	37	18 (49)
Respiratory rate < 9/min	0	0 (0)
Respiratory rate > 25/min	5	4 (80)
*S*po_2_ < 90%	0	0 (0)
Intubated/attempted intubation	14	4 (29)
Obvious massive haemorrhage	1	1 (100)
Systolic blood pressure < 90 mmHg	0	0 (0)
Physiology total	75	31 (41)
Anatomy		
Facial injury with risk for airway obstruction	7	4 (57)
Flail chest	2	1 (50)
Suspected pneumothorax	21	9 (43)
Stab or gunshot wound proximal to knee or elbow	10	7 (70)
Suspected pelvic fracture	10	7 (70)
Crushed, mangled or amputated extremity	2	1 (50)
Two or more long bone fractures	4	1 (25)
Open fracture with ongoing haemorrhage	0	0 (0)
Open skull fracture or impression fracture	2	1 (50)
Suspected spinal cord injury	14	11 (79)
Burn injury > 15% total body surface area	2	2 (100)
Anatomy total	74	44 (59)
Other		
Several severely injured (suspected or	14	8 (57)
confirmed)		
Two or more criteria for reduced trauma	8	6 (75)
team are fulfilled		
Reduced team finds perceived stable	5	1 (20)
patient unstable		
Other total	27	15 (56)
Undocumented criteria	53	33 (62)
Full team total	229	123 (53·7)
Reduced team		
Co-morbidity		
Age > 60 years	9	7 (78)
Age < 6 years	7	6 (86)
Severe co-morbidity	8	4 (50)
Pregnancy	0	0 (0)
Increased risk for haemorrhage	4	2 (50)
Co-morbidity total	28	19 (68)
Mechanism of injury		
Co-passenger dead	1	1 (100)
Entrapped person	4	3 (75)
Ejected from vehicle/motorcycle	27	23 (85)
Pedestrian, cyclist run down at > 30 km/h	33	28 (85)
or thrown in the air		
Collision speed > 50 km/h	61	61 (100)
Deformed vehicle compartment	8	8 (100)
Airbag set off	14	14 (100)
Vehicle roll-over	8	8 (100)
Fall > 5 m (adults)	27	17 (63)
Fall > 3 m (children)	5	5 (100)
Mechanism of injury total	188	168 (89·4)
Undocumented criteria	70	55 (79)
Reduced team total	286	242 (84·6)

Values in parentheses are percentages. RTS, Revised Trauma Score; GCS, Glasgow Coma Scale. *S*po_2_, oxygen saturation measured by pulse oximetry.

### Mortality

No deaths were registered in patients triaged to reduced teams. Median time from activation of reduced team to full team upgrade for the five affected patients was 11 (range 0–21) min. Median NISS was 17 (range 6–50), and one upgraded patient died. There were 12 deaths among undertriaged patients, eight (4·8 per cent) in the ‘before’ and four (11 per cent) in the ‘after’ study period (*P* = 0·229). The median age of patients who died was 80 (range 66–90) years and median NISS 46 (range 27–59). All had falls. For the total population of included patients, the *W* statistic (excess survivors per 100 patients compared with TRISS model predictions) did not change significantly: 2·123 (95 per cent c.i. 1·070 to 3·177) ‘before’ *versus* 2·510 (1·127 to 3·892) ‘after’.

## Discussion

The present study found that the introduction of a formalized TTA protocol with a two-tiered response was associated with reduced undertriage and increased overtriage. Trauma team resource consumption was significantly reduced. For the study period as a whole, increasing age and falls increased risk for undertriage and decreased risk for overtriage, whereas motor vehicle-related accidents showed the opposite effects.

Triage precision before implementation of the TTA protocol was poor. Informal activation of trauma teams did not correctly identify victims of major trauma. A relative reduction in overall undertriage of 32·6 per cent followed system revision. The current undertriage rate of 19·1 per cent is still considered unacceptable and continued efforts to further improve triage precision are essential. The death of one upgraded patient with an NISS of 50 emphasizes that the practice of upgrading a reduced team to a full team requires constant monitoring. There was a highly significant 41·2 per cent relative reduction in undertriage in prehospital responses without an anaesthestist but only a non-significant trend towards less undertriage when an anaesthetist was present. When studied in the logistic regression model, prehospital responses involving an anaesthetist had a higher overall triage precision with reduced risk for undertriage as well as overtriage. In the Norwegian prehospital system, anaesthetist-manned units normally attend patients considered severely injured by either dispatch or paramedic-manned units already at the scene, whereas paramedics respond to a considerably less preselected patient population. Direct comparison between the two EMS provider categories was therefore considered both unreasonable and counterproductive.

This undertriage rate in responses without an anaesthestist remains high, but is also seen in other organized trauma systems^5^, ^10^, ^12^. Initiatives such as increasing the number of employees with a certificate of competence in prehospital care have been launched to improve quality of care, but further studies on the reasons for undertriage are called for^37^. Triage precision should also be addressed in responses with an anaesthetist, although an undertriage rate of 5–10 per cent is considered acceptable^22^.

All 12 patients who died in the undertriaged group were over 66 years old and had falls. The logistic regression model showed that increasing age and falls were both found to increase risk for undertriage and decrease risk for overtriage. Velmahos *et al.*^38^ have previously found that unintoxicated patients over 55 years of age with low-level falls had a high likelihood of significant injuries. Others have recommended that age over 69 years should be a criterion for TTA^39^ or a need for enhanced focus on apparently low-impact injuries in this population^5^.

It was expected that a reduction in undertriage would be accompanied by increased overtriage. Although TTA is beneficial for trauma victims, it may lead to suboptimal care for other patients^40^. The two-tier TTA system was designed to reduce excess resource consumption due to overtriage. Skilled hours spent on overtriage per major trauma victim, reflecting the exploitation of manpower on minor trauma cases, were reduced from 7·4 to 7·1 after implementation of this system. This is of particular interest given the current focus on improvement of quality and cost reduction in healthcare.

Much emphasis has been put on mechanism of injury as a criterion for TTA^1^, as it can contribute to the effectiveness of the triage tool in the absence of changes in vital signs or obvious anatomical injury^41^. Consequently, the findings that motor vehicle-related accidents were associated with both reduced risk for undertriage and increased risk for overtriage were expected. It was alarming, however, to find that falls carried an odds ratio for undertriage of 2·46. Educational efforts are obviously needed to reduce undertriage in this patient group.

The present study has a number of limitations. The ‘before’ study period involved a review of trauma registry data restricted to variables already defined in the trauma registry. Missing documentation of TTA criteria remained a challenge throughout the study period. A short 18-month ‘after’ period compared with a 60-month long ‘before’ period increases the risk for type II errors. The study is also susceptible to the Hawthorne effect^42^. The simultaneous introduction of revised TTA criteria and the two-tiered response also complicated the evaluation of the study outcome. Even though major trauma defines the threshold against which triage protocols are tested, several conflicting definitions exist^43^. An NISS of over 15 was used to define major trauma and adhere to the inclusion criteria recommended by the Utstein template for uniform reporting of trauma data^28^. This implies that undertriaged patients were those included in this group who were not met by a full or reduced trauma team. In contrast, Curtis *et al.*^44^ considered all patients with an ISS of more than 15 assessed by a trauma standby (similar to the SUH reduced team) to be undertriaged. The different definitions highlight the difficulties of comparing data. The way in which definitions of major trauma influence calculations of triage precision merit investigation.

Implementation of system revisions can be a challenging enterprise with over 250 barriers identified in the literature^45^. To improve implementation of the new TTA criteria a teaching programme was developed addressing specific aspects of system revision. The programme was included in hospital and prehospital educational outreach visits arranged by trained instructors, a periodical newsletter was published and information posters were designed to remind staff of the new system for tiered TTA. To reduce the impact of failures related to lack of experience with the protocol, all patients from the 6-month implementation phase were excluded. However, examples of misapplication of the triage protocol were found throughout the entire ‘after’ period and act as reminders that implementation is a continuous process.

Converting from an informal one-tiered TTA to a formalized two-tiered TTA lowered the threshold for immediate access to high-quality trauma care by reducing undertriage rates. Although the introduction of a reduced trauma team increased the overtriage rate, the number of work hours spent per major trauma victim was reduced.

## Collaborators

The members of the Rogaland Trauma System Study Collaborating Group were Espen Fevang, Kjetil Søreide, Eldar Søreide, Johannes Lokøy, Pieter Oord, Carina Lavransdatter Fossåen, Pål Stokkeland and Kristian Strand.
